# Teachers' characteristics predict students' guidance for healthy lifestyle: a cross-sectional study in Arab-speaking schools

**DOI:** 10.1186/s12889-022-13795-5

**Published:** 2022-07-26

**Authors:** Rachel Wilf-Miron, Roaa Kittany, Mor Saban, Ilya Kagan, Mor Saban

**Affiliations:** 1 Department of Health Promotion, School of Public Health, Sackler Faculty of Medicine, Tel Aviv University, Tel Aviv, Israel; 2 Gertner Institute for Epidemiology and Health Policy Research, Sheba Medical Center, Ramat Gan, Israel; 3 Nursing Department, School of Health Sciences, Ashkelon Academic College, Ben Tzvi 12, 78211 Ashkelon, Israel

**Keywords:** Teacher, Health behaviors, Burnout, Organizational commitment, Job satisfaction student guidance

## Abstract

**Introduction:**

Schools are valuable settings for implementing healthy lifestyle interventions. Teachers' health behaviors affect their health and well-being and might affect their position as role models for students. This study aimed a) to assess health behaviors, health perceptions, burnout, job satisfaction, and organizational commitment among Arab school teachers in Israel; b) to examine the relationship between these variables; and c) to explain the variance of healthy lifestyle promotion among students.

**Methods:**

A cross-sectional study using a structured questionnaire was conducted among 150 teachers (mean age 39 years, 85% women) in May-June 2020.

**Results:**

Most respondents (79%) were overweight and obese, 79% reported unhealthy nutrition and not reaching the recommended physical activity target, 47% slept >7 hours at night and 54% defined their health status as very good or excellent. Reported burnout levels were high. Organizational commitment and job satisfaction were high while students’ guidance towards a healthy lifestyle was moderate. Burnout was negatively correlated with health perception, organizational commitment, and job satisfaction. Health perception was positively correlated with organizational commitment, job satisfaction and promoting a healthy lifestyle among students. Logistic regression analysis revealed that job satisfaction, performance of PA according to the recommendations and burnout predicted 51% of the variance of healthy lifestyle promotion among students.

**Conclusions:**

Teachers in Israeli Arab schools report unfavorable health behaviors and health perception as well as high burnout levels. The findings suggest implementing intervention programs to reduce teacher burnout and creating organizational conditions that would encourage teachers to adopt a healthy lifestyle and help them promote healthy lifestyle habits among their students.

**Supplementary Information:**

The online version contains supplementary material available at 10.1186/s12889-022-13795-5.

## Highlights

Teachers' health behaviors may affect their position as role models for students.

Israeli Arab teachers had unfavorable health behaviors and high body mass index.

Burnout inversely correlated with organizational commitment and job satisfaction.

Better perceived health predicted lower burnout and higher job satisfaction.

Improved school environment could improve teachers' wellbeing and role modeling.

**Supplementary Information:**

The online version contains supplementary material available at 10.1186/s12889-022-13795-5.

## Introduction

Children spend a considerable part of their waking hours at school, therefore, schools are valuable settings for implementing healthy lifestyle interventions, such as promoting healthy eating and physical activity, aimed at preventing childhood obesity [1]. School-based interventions are accessible to all children, regardless of their parents' time or ability to pay, and are therefore implemented in an equitable manner.

The teacher’s job is to educate, mentor and outline students’ values, in addition to providing knowledge. Teachers' health behaviors affect their health and well-being and might affect their position as role models for students. A Chinese study among 4-6-grade students and their teachers found that teachers’ health awareness, positive health attitudes, never-smoking and regular-exercise were positively associated with healthy eating behaviors among their students (having breakfast, eating vegetables and dairy products every day), and negatively associated with unhealthy behaviors (daily intake of fried foods, desserts and sugary beverages) [2]. A cross-sectional study among 4,010 adolescents aged 12-17 years in California demonstrated that respondents with a teacher-identified role model were the most likely to engage in health-promoting behaviors (adequate fruit and vegetable consumption and regular physical activity) as compared with adolescents without a role model. Teens who reported a teacher as a role model were far less likely to engage in most health risk behaviors such as smoking [3]. Health behaviors of Israeli students are of special concern since teenage students (11, 13 and 15-year-olds) had worse eating habits and lower physical activity scores then their counterparts in other studies [4].

Organizational commitment is characterized by the willingness of employees to be attached to the organization, their identification with the organization, and the longevity of their membership [5]. To accomplish their purpose, schools rely on individual teachers with high levels of motivation and commitment to their workplace. In addition to being required to take frequent courses to maintain their professional knowledge, teachers’ roles require responsibility, empathy and coping with diverse difficulties of students. However, teachers frequently face inadequate societal and parental appreciation and support, leaving them to feel alone in their battle behind the classroom door [6]. This may lead to higher burnout and lower work satisfaction. Burnout is classified as an occupational phenomenon resulting from chronic workplace stress that has not been successfully managed. It is characterized by three dimensions: feelings of energy depletion or exhaustion; increased mental distance from one’s job, or feelings of negativism or cynicism related to one's job; and reduced professional efficacy [7]. A systematic review and meta-analysis of 45 articles and 49 independent samples (*N* = 14,410) demonstrated high levels of burnout among secondary school teachers: 28% suffered from severe emotional exhaustion, 38% reported high levels of depersonalization and 40% reported low levels of personal accomplishment [8].

Burnout can provoke diverse problems that may affect teachers, students, and institutions. For example, burnout levels were inversely related to job satisfaction among Czech primary school teachers [9]. Perceived stress related to workload and sense of teaching efficacy were directly related to perceived job satisfaction among 664 primary and secondary school teachers from British Columbia and Ontario, Canada [10].

The motivation to study the associations between burnout, organizaitonal factors and health behaviors stems from previous studies: In a large national study among health workers in Israel, participants named work overoad, difficulties to maintain work-life balance, and too many administrative tasks as the leading factors contributing to burnout [11]. Among phyisians, negative association was found between job stress and perceived health status, which in turn was positively correlated with physical activity, healthy eating behavior and normal Body Mass Index [12]. Emotional exhaustion (a componenet of burnout) was positively associated with higher fast food consumption and negatively associated with exercise frequency among physicians and nurses in several European countries [13]. These studies among health professionals point to a possible pathway to reduce burnout by promoting a healthy lifestyle among other professionals, too.

Among Greek teachers, lower emotional exhaustion was associated with greater job satisfaction [14]. Negative relationship was found in Turkey between teachers' burnout and their organizational commitment [15]. Higher participation in leisure-time physical activity (PA) was associated with a more positive perceived health among Flemish secondary school teachers [16]. Integrating short aerobic activity (10 minutes of less) bouts into organizational routine in everyday life of schools or worksites was found effective at increasing social support for physical activity within community settings [17]. A recent systematic review demonstrated strong positive effects of workplace initiatives on weight-related outcomes, mental health, and musculoskeletal health [18].The Israeli Arab population, which accounts for 21% of the 9.1 million inhabitants of the country, is characterized by lower socioeconomic ranking, and higher prevalence of obesity, diabetes, hypertension and cardiovascular disease compared with the Jewish population [19]. A national study among 12-18 old school children demonstrated higher prevalence of overweight and obesity among Arab, compared with Jewish students [20]. Given the importance of health behaviors in general, and in school-aged children from vulnerable populations specifically, this study aimed a) to assess health behaviors, health perceptions, burnout, job satisfaction, and organizational commitment among Arab school teachers in Israel; b) to examine the relationship between these variables; and c) to explain the variance of healthy lifestyle promotion among students.

## Methods

### Study setting and participants

A cross-sectional study was performed among primary and high-school teachers working in Baqa al Gharbiah, a city in central Israel inhabited by 30,000 Muslim Arab residents. Out of 15 schools in the city, a convenience cluster sample of eight schools from different municipal areas was selected for the study. The principals of 8 schools were contacted, and 7 of them (5 primary schools and 2 secondary schools) agreed that their teachers participate in the study. A sample size of 113 was calculated using the online WinPepi software [21] according to the following parameters: 90% power, *r*=0.3, and α=5%.

After receiving ethical and administrative approvals, one of the researchers (RK), was invited by the principal of each of the 7 participating schools to attend the school staff meeting. After being introduced by the principal, she provided an explanation about the study and its goals, clarifying that teachers have the right to refuse to participate. Teachers who agreed to participate were asked to complete the questionnaire, which required about 20 minutes for completion. Teachers were recruited on a "stand-by" basis, volunteers (not invited to staff meetings) and those on maternity or sick leave did not participate in the study. The data were collected during May and June 2020.

All methods were carried out in accordance with relevant guidelines and regulations or Declaration of Helsinki.

### Research tool

#### The questionnaire consisted of six sections

***Socio-demographic and professional information*** included gender, age, family status, type of school, percent of appointment e (i.e. percent of full time equivalent), and tenure position. One question addressed the presence or absence of chronic disease (diabetes, hypertension or cardiac disease).

***Health perception*** was measured using the question: "How is your health in general?” which is widely used to assess general health in various populations and is a well-known predictor of health outcomes [22]. The participants were asked to rank their answers on a Likert scale ranging from 1 (poor) to 5 (excellent).

***Health behaviors*** were measured by items and sub-scales used in previous surveys among Israeli physicians [12] and nurses [23]. *Achieving the physical activity (PA*) *target* was defined as accumulating 150 weekly minutes of medium-intensity PA, 75 minutes of vigorous-intensity PA or combination of medium-intensity and vigorous intensity, in a typical week. This target is based on updated guidelines, stating “Adults should do at least 150 minutes to 300 minutes a week of moderate-intensity, or 75 minutes to 150 minutes a week of vigorous-intensity aerobic physical activity, or an equivalent combination of moderate- and vigorous-intensity aerobic activity”. Considering the modest (i.e. minimal) requirement, teachers had to accumulate 150 minutes of medium-intensity PA, with each minute of vigorous-intensity PA translated to 2 minutes of medium-intensity PA [24, 25]. For example, a teacher who accumulates 30 weekly minutes of running (equivalent to 60 minutes of medium-intensity PA) and takes yoga classes for 100 weekly minutes, accumulates 160 minutes of medium intensity PA per week, i.e. achieves the recommended PA target.

*Nutrition and eating habits* were measured by the intake frequency of 4 items - eating breakfast, consuming a Mediterranean diet [26], consuming processed foods, and drinking sugar-sweetened beverages - that were ranked on a 5-point Likert scale ranging from never (1) to daily/almost every day (5). Additional items measured the daily intake of glasses of water, and the number of fruit and vegetable servings consumed. Consumption of breakfast and Mediterranean diet every day or almost every day, consumption of sugar-sweetened beverages or processed food less than once a week or never, consumption of ≥6 water glasses (the Mediterranean diet recommends 6-8 water glasses per day) [26] and ≥5 servings of vegetables and/or fruit per day [27] was considered healthy. Healthy nutrition was defined as accumulating 5 or 6 of those healthy items. This cut point was based on a questionnaire, developed and validated for a large physician study [12]. Seven nutrition items in the physician study were modified to six, by omitting the question on lunch, which is less relevant to the Israeli teachers’ workday. Adaptation of the “healthy score” to the current study, yielded the 5 or 6, out of 6 items, constituting a “healthy nutrition”.

*Sleep* was measured by the number of hours slept at night, presented as four categories - ≤5, 6, 7, and ≥8 hours. *Compliance with screening for early detection of risk factors and diseases* was measured by the question "Do you undergo the periodic age-recommended screening tests?" The responders could choose among "not at all"; "partially" and "fully as recommended".

***Organizational commitment*** was measured using the Hebrew version of the 15-item Porter's Organizational Commitment Questionnaire (OCQ) [28]. The questionnaire examines teachers’ adherence on a scale of 1 (very low) to 7 (very high) to the organization's goals and values through their willingness to invest efforts for the organization's success. A mean score was calculated for each participant, with higher mean scores representing higher organizational commitment. The reliability of the tool (Cronbach’s alpha) in the current study was 0.78.

***Burnout*** was measured using the 14-item Teachers' Burnout scale [29]. Scale is composed of 3 sub-scales: An overwhelming exhaustion (items 1,2,6,8,12); a sense of lack of accomplishment (items 4, 7, 9, 10, 14) and depersonalization, i.e. feelings of cynicism and detachment from the job (items 3,5,11,13) [29]. Participants were asked to rank sentences representing those burnout domains on as scale from 1 (never) to 6 (always). A mean score was calculated, with higher mean scores representing higher burnout. Cronbach’s alpha was 0.93.

***Job satisfaction*** was measured using a 14-item tool [30]. The participants were asked to rank their job satisfaction on a scale from 1 (low satisfaction) to 6 (high satisfaction). A mean score was calculated for each participant with higher scores representing greater job satisfaction. Cronbach's alpha of this tool was 0.92.

***Promoting healthy lifestyles among school students*** was measured by tool constructed by the authors following a literature review. The tool consisted of 14 statements representing activities expected of teaches to promote health behaviors and healthy lifestyle among their students. Teachers ranked the degree of activity execution on a scale of 1 (to a very small extent) to 5 (to a very large extent). Higher mean scores represented greater health promotion among students. Cronbach's alpha of this tool was 0.91.

To validate the whole questionnaire, it was administered to an expert panel of five teachers specializing in school-based health promotion who provided feedback on the usefulness and clarity of the questionnaire for Arabic-speaking teachers. Following their feedback and prior to data collection, a pilot study was conducted among 10 teachers. The teachers were asked to review the questionnaire and to clarity the statements and wordings. Consequently, a few terms that were deemed complicated (such as satisfaction, climate and exhaustion) were translated to Arabic and added to the questionnaire. The teachers that comprised the expert panel and those that participated in the pilot study were employed at schools located in the same city that did not participate in the study.

### Statistical analysis

Continuous variables were summarized as mean and standard deviation. Categorical variables were summarized as frequencies (number of cases and percentages). The Mann-Whitney test was performed to compare between the two groups. To allow nonparametric analyzes and logistic regression, the study variables (burnout, organizational commitment, and job satisfaction) were recoded into dichotomous scales (high versus low). The recoding process was based on a Median Split method for turning an order scale variable into a categorical one. Firstly, we computed the median of these variables. Secondly, all the values below the median were defined as a “Low” category and all values above were determined as a “High” category. In the variable health perception, excellent and very good perceptions were categorized as “high” or “healthy”. The effect of age was analyzed by four age groups (21-32, 33-39, 40-45, and 46-59 years). Since age failed to demonstrate a normal distribution, the cut-off points were defined by age quartiles, as follows: percentile 25 = 32.00; percentile 50=39.00, percentile 75=45.0.

Spearman correlation coefficients were calculated to determine associations between ordinal variables such as the organizational commitment, burnout, satisfaction and guidance towards a healthy lifestyle. Multiple regressions were performed to examine the contributions of independent variables to explaining the dependent variables. These were significant in univariate models (up to a threshold of *p* <0.2). A *p* value <0.05 was considered statistically significant. Data were analyzed using SPSS 26.0 (IBM Corporation, Armonk, NY, USA).

## Results

**Fig. 1:**
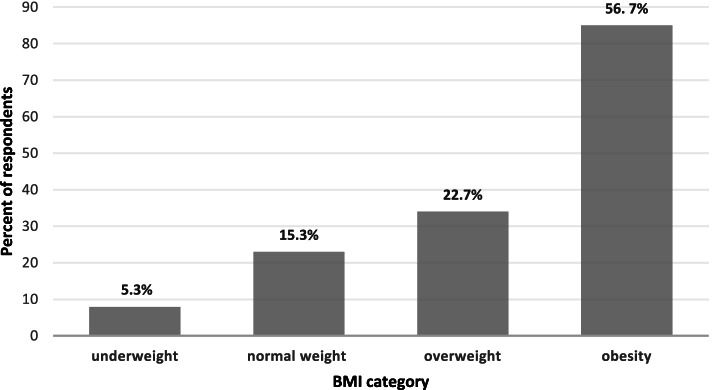
Distribution of the study population by BMI categories (*n*=150)

Of 185 teachers approached, 150 agreed to participate and completed all parts the questionnaire (a response rate of 81%). The respondents’ sociodemographic characteristics are shown in Table 1. The mean age of respondents was 38.6 years (SD=8.7; range 21-59); 85.3% were women and 87.3% were married. Respondents reported a mean of 13.8 years of teaching experience (SD=8.13; range 1-35), and two-thirds of them worked in primary schools. The mean body mass index (BMI) of the study population was 33.74 (SD = 11.18); most of the population (79.34%) were overweight or obese (Fig. 1). Most male teachers (96%) were obese compared to 50% of female teachers (*p*<0.05). A significantly higher percentage of married teachers were obese compared with unmarried ones (60% vs. 30%, *p*<0.05). Over half of the respondents (54%) defined their health status as very good or excellent. Teachers who perceived their health as very good or excellent were less likely to be obese compared with those who reported good, fair, or poor health (72% vs 87%, respectively, *p*<0.05). Nine percent of respondents reported chronic morbidity. Most respondents who reported having a chronic disease were also obese (93%) compared to 53% of those without chronic disease (*p*<0.05).

**Table 1:** Participants' sociodemographic, professional and health characteristics

		Study participants *N*=150	
Characteristics	Category	N	(%)
Gender	Male	22	(14.7)
	Female	128	(85.3)
Age	21-32	38	(25.3)
	33-39	49	(32.7)
	40-45	29	(19.3)
	46-59	34	(22.7)
Family status	Married	131	(87.3)
	Single, divorced or widower	19	(12.7)
Type of school	Primary	98	(65.3)
	Secondary school	52	(34.7)
Appointment percent^a^	75% or less	43	(28.6)
	76% and more	107	(71.3)
Tenure status	Yes	116	(77.3)
	No	34	(22.7)
Chronic disease	Yes	14	(9.3)
	No	136	(90.7)

^a^32 teachers (21.3%) worked less than 50% of an appointment; another 11 (7.3%) worked between 50 and 75%. Those two categories were combined

Most participants reported unfavorable health behaviors (Table 2). Most respondents (79%) did not reach the recommended PA target and 27% of them did not exercise at all. Male teachers reported performing more PA compared to female teachers (46% v 16%, *p*<0.05), unmarried teachers performed more PA than married teachers (42% v 18%, *p*<0.05) and secondary school teachers – more than primary school teachers (31% v 15%, *p*<0.05). Most respondents (79%) also had unhealthy eating habits. Older teachers reported better nutrition habits: 42% of 46-59-year-old teachers had healthy eating habits compared with 19% of 21-32-year-olds (*p*<0.05).

**Table 2:** Health behaviors and health perceptions among teachers

		Study participants *N*=150	
Health Behaviors	Category	n	(%)
Perceived health status	Excellent	27	(18.0)
	Very good	54	(36.0)
	Good	54	(35.3)
	Fair	13	(8.7)
	Poor	3	(2.0)
Healthy nutrition	Yes	31	(20.7)
	No	119	(79.3)
Physical Activity (minutes/week)	0 min	41	(27.3)
	1 to 74.9 min	53	(35.3)
	75 to 149.9 min	25	(16.7)
	150+ min	31	(20.7)
Sleeping (hours/day)	≤6	70	(46.7)
	7 and more	80	(53.3)
Performing age-recommended screening tests	No	30	(20.0)
	Partially	92	(61.3)
	Fully as recommended	28	(18.7)

Almost half of respondents slept less than seven hours a day. A higher percentage of 21-32-year-old teachers (71%) reported adequate sleep hours (7 hours) compared with 38% of 46-59-year-olds (*p*<0.05). Only one fifth fully performed screening tests as recommended.

Respondents reported high mean burnout (M=3.14; SD=1.09, 1-6 scale), moderate-high mean organizational commitment (M=5.27; SD=0.83, 1-7 scale) and moderate mean job satisfaction (M=4.35; SD=0.94, 1-6 scale). Secondary school teachers were more committed to the organization compared with primary school teachers (60% vs. 42%, *p*<0.05) and more satisfied with their work (62% vs. 42%, *p*<0.05).

The respondents reported moderate healthy lifestyle promotion among their students (M=3.41, SD=0.76; 1-5 scale). More than half of the teachers (62%) believed that it is the teachers’ task to promote healthy lifestyle habits among their students, however less than half (46%) believed that their guidance is effective. Most teachers (70%) reported that their students expect them to serve as role model for health behaviors, however 53% believe that work conditions do not allow for such a guidance.

The relationship between health behaviors and the study variables is demonstrated in Table 3. As shown, burnout was higher among the respondents who did not meet the recommended target of physical activity, compared with those who met the PA target. Similarly, organizational commitment and job satisfaction were significantly higher among teachers who met the recommended target of physical activity compared with those who did not meet the target. Job satisfaction was significantly higher among those consuming a healthy diet compared to those consuming an unhealthy diet. No direct association was found between other health behaviors and the study variables.

**Table 3:** Differences in burnout, organizational commitment and job satisfaction by health behaviors (Mann-Whitney U)

	Burnout		Organizational commitment		Job satisfaction	
	*Chi*^*2*^	*P*	*Chi*^*2*^	*P*	*Chi*^*2*^	*P*
Healthy nutrition	1425.50	0.052	2173.00	0.127	2354.00	0.018
Physical activity	1090.00	<0.001	2314.00	0.029	2398.50	0.010
Sleeping hours	2977.00	0.505	2638.00	0.541	2662.00	0.603
Age-recommended screening tests	1707.50	0.664	1599.50	0.346	1646.000	0.469

*Notes*: The median scores that were used to define “low” and “high” categories were as follows: Organizational commitment - 5.2667; Job satisfaction - 4.4286; Burnout - 3.0714

Burnout was negatively correlated with health perception, organizational commitment, job satisfaction and promoting a healthy lifestyle among students (Table 4). Health perception was positively correlated with organizational commitment, job satisfaction and promoting a healthy lifestyle among students.

**Table 4:** Spearman correlations between the main study variables *(n=150)*

Variables	1	2	3	4
1. Health perception	-----			
2. Organization commitment	.32**	------		
3. Burnout	-.45**	-0.52**	------	
4. Job satisfaction	.45**	0.56**	-0.63**	------
5. Guiding for healthy lifestyle	.37**	0.28**	-0.52**	0.57**

**p < 0.001

Logistic regression analysis, utilized all the variables that were significant in the univariate analyses: age, gender, health perception, nutrition, PA, burnout, organizational commitment and job satisfaction. he analysis revealed that three of them - job satisfaction, performance of PA according to the recommendations and burnout - predicted 51% of the variance of healthy lifestyle promotion among students (Table 5).

**Table 5:** Logistic regression of predictors of healthy lifestyle promotion among school students

Variables	Category	OR	95% CI		*P*
Age	21-32 years				.974
	33-39 years	.85	.270	2.71	.788
	40-45 years	1.03	.28	3.87	.962
	46-59 years	1.15	.28	4.726	.850
Gender	female vs. male	3.85	.82	18.05	.087
Health perception	healthy vs. non-healthy	1.89	.69	5.19	.216
Nutrition	healthy vs. non-healthy	.86	.27	2.77	.802
Physical activity	healthy vs. non-healthy	6.39	1.77	23.07	.005
Burnout	High vs Low^a^	.34	.13	.89	.028
Organizational commitment	High vs Low^a^	.83	.33	2.10	.699
Job satisfaction	High vs Low^a^	7.57	2.91	19.64	.000

*CI* confidence interval, *OR* odds ratio

*Notes*: Independent variables entered - age, gender, health perception, nutrition, exercise, burnout, organizational commitment, job satisfaction; *R*^2^ = 0.505 (51%)

^a^Notes: The median scores that were used to define “low” and “high” categories were as follows: Organizational commitment - 5.2667; Job satisfaction - 4.4286; Burnout - 3.0714

## Discussion

This study aimed to discover teachers' health behaviors and perceptions, their associations with burnout, job commitment and job satisfaction as well as the contribution of these variables to the prediction of promoting healthy lifestyles among school students. The results of the current study allow to draw a potential framework that could explain the relationship between the study variables: Teachers with better perceived health reported higher organizational commitment, job satisfaction and promoting a healthy lifestyle among students. Teachers with higher burnout, perceived their health as worse, were less committed to the school, less satisfied with their work and promoted a healthy lifestyle among their students to a lower extent.

Those findings are supported by previous studies [9, 16, 18, 31, 32]. In another study, health perception was correlated with health status [33], which was largely determined by health behaviors. Nurses' Health Study pointed to the importance of health behaviors, demonstrating that healthy nutrition, performing PA regularly, not smoking and maintaining a normal BMI can prevent 55%, 72% and 44% of deaths related to all causes, cardiovascular disease and cancer, respectively [34].

The study revealed unfavorable health behaviors and health perception as well as high burnout levels among primary and high-school teachers working in schools with an Israeli Arab population. The teachers had a high prevalence of overweight and obesity, with 79% reporting BMI≥25. These figures are considerably higher compared with those of a sample of the general Israeli population of similar ages (57% reported a BMI≥25) [35] and with a sample of teachers in Brazil (47% reported BMI≥25) [36]. A nation-wide study among French educational staff revealed lower health risk-behaviors, like smoking, among teachers, compared with non-educational employees. Results were more mixed regarding the prevalence of overweight or obesity [37].

Only one-fifth of respondents underwent age-recommended screening tests compared to one-third of Israeli nurses who participated in a large national study reported undergoing age-recommended screening tests [23]. Low utilization of screening tests have been documented among the Israeli Arab population, for example rates of ever screening for colorectal cancer were twice higher among Jewish, compared with Arab adults [38].

Slightly more than half of respondents (54%) perceived their health as very good or excellent, compared with 67% of a sample of the general Israeli population [39]. This figure might be explained by the very high prevalence of obesity among respondents since obesity was associated with worse health perception.

Only 21% of respondents (46% and 16% of males and females, respectively) achieved the recommended PA target. This finding is in accordance with lower rates of PA among adult Arabs compared with Adult Jews in Israel (17% and 35%, respectively), and especially low rates among Arab females, compared with Arab males (16% and 27%, respectively) [40]. The German Gutenberg Health Study Cohort revealed a healthier lifestyle regarding physical inactivity, especially among male teacher, compared with other professionals [41]. A study conducted among 978 primary and secondary school teachers in Brazil revealed that 71.9% of respondents did not achieve the PA recommended target. Not being physically active was associated with occupational factors such as bad work-life balance. Insufficient PA was associated with female gender and with higher levels of BMI. Being physically active was correlated with very good of excellent health perception [42].

Only one-fifth of respondents reported healthy eating habits, an unfavorable finding that is comparable with the rate reported by Israeli physicians (16.7%) .

The high burnout levels (3.14 on a 1-6 scale) reported in the current study are higher than those previously reported among teachers in a similar Arab city (2.79) [43] and also higher compared with an average score of 3.4 on a 1-7 scale a national study among healthcare workers [44].

Satisfaction with the teaching job was rather good (4.35 on a 1-6 scale), but lower than that reported by teachers in national studies, were 89% and 92% of the Arab and Jewish teachers, respectively, were satisfied with their profession [45]. Commitment to the school was reportedly high (5.2 on a 1-7 scale), like other studies among Arab teachers in Israel [46, 47]. Good job satisfaction and high organizational commitment in the presence of high burnout levels is different from the associations described in the literature, for example, all 3 dimensions of burnout were negatively associated with job satisfaction among teachers in Jammu, India [10, 48]. A study among 745 Israeli teachers from 98 primary, middle and high schools in Israel found that, female teachers’ level of satisfaction was significantly higher compared with male teachers. Jewish teachers expressed a higher level of satisfaction than the Arab teachers, but this difference was not statistically significant [49]. However, our study did not find gender difference in teachers’ satisfaction.

Respondents reported good levels of healthy lifestyle promotion among their students. The majority of the teachers believed that it is the teachers’ task to promote healthy lifestyle habits among their students, however less than half believed that their guidance is effective. Despite students’ expectation that teachers would serve as role model for health behaviors, work conditions frequently do not allow for such a guidance. Similarly, most of the teachers in a large city in the USA believed that they had to act as role model for students regarding healthy nutrition, however they were less confident in their ability to teach healthy nutrition in class [50].

Children and adolescents with healthy lifestyle habits have lifelong healthier behaviors, as was demonstrated for physical activity, for example [33]. Teachers are in a unique position to foster health promotion because they have direct access to large numbers of children. Therefore, there is great importance to create the environment that would help teachers to fulfill their position as a role model for healthy lifestyle, through appearance, practice and goal setting [51]. Teachers’ sub-optimal health behaviors and health perception might be an obstacle to this important role model position. This is true for the entire population and even more so for the Arab society who demonstrated unfavorable health behaviors and worse health outcomes, compared with the general Israeli population [19, 52].

One should bear in mind that the study took place during May-June 2020, in the first year of the COVID-19 pandemic. Vast changes were imposed on the entire population, including school students and educational staff. The current study took place between the first and second national lockdowns, with resumption of physical learning. However, public health measures like physical distancing, face mask wearing, and hand hygiene influenced school routines. Both teachers and students were exposed to the stress related to the fear of contracting the infection (before the development and approval of COVID-19 vaccines), family members and friends’ job loss or the mandate to avoid large gathering in times of joy and grief. The pandemic had an effect of health behaviors of the general population: A survey among Israeli adults demonstrated that more people stopped engaging in routine PA compared with those who began exercising during the lockdown. A higher percentage of people reported weight gain than those who reported weight loss [53]. The pandemic also had an effect on the health of children and adolescents, pointing to even greater importance of student guidance to lead a healthy lifestyle: In a large (N=37K) retrospective cohort study in Israel, the pandemic correlated with overall weight gain among children and adolescents [54]. A study among US teachers during October 2020 revealed a high level of average burnout stress score. COVID-19 anxiety, current teaching anxiety, anxiety communicating with parents, and administrative support were predictors for burnout-stress [55]. The study includes several limitations. Since the study utilized a convenience sample of school principals who agreed to participate (seven out of 8 schools) and of teachers working in the participating schools who agreed to complete the questionnaires (150 out of 185 who were given questionnaires), it is possible that principals who agreed to participate in the study knew that their school was more health promoting and teachers who agreed to participate might have been more aware of the health promoting agenda. Therefore, the results of the study might be an under-representation of the actual problem. Also, it is hard to generalize the results of the study (based on a convenience sample of 150 teachers working in a single Arab city) to the broader teaching population, whether Arab or the general teaching population, in Israel. Although the questionnaires were completed anonymously, a social desirability bias may also have led to under-representation of the actual problem as teachers may have under-reported health-related issues. In addition, the questionnaire was in Hebrew, which is not the first language of the study respondents. However, the teachers’ university training and routine work require them to master Hebrew at a very good level. In addition, following the pilot study, words that were deemed difficult to understand were translated to Arabic and added to the questionnaire. The study took place in May-June 2020, during the COVID-19 pandemic. Although schools were open, they functioned under strict social distancing measures, which may have affected teachers’ perceptions, making it difficult to generalize the results of the study to "normal" times [56].

## Conclusions

The study revealed unfavorable health behaviors and health perception as well as high burnout levels among teachers from seven Arab-speaking schools. The findings constitute a risk for teachers’ physical and emotional well-being and may impair their ability to act as role models and promote healthy habits among their students. The findings are a call to action for the educational system to create conditions that would encourage staff to adopt a healthy lifestyle, implement intervention programs to reduce teacher burnout and help them guide students towards a healthy lifestyle.

## Supplementary Information

**Additional file 1.**

## Data Availability

The data that support the findings of this study are available from the corresponding author, upon reasonable request.

## Abbreviations

PA: Physical activity
BMI: Body Mass Index
