# Correction: Teachers’ characteristics predict students’ guidance for healthy lifestyle: a cross-sectional study in Arab-speaking schools

**DOI:** 10.1186/s12889-022-13902-6

**Published:** 2022-08-17

**Authors:** Rachel Wilf-Miron, Roaa Kittany, Mor Saban, Ilya Kagan

**Affiliations:** 1 Department of Health Promotion, School of Public Health, Sackler Faculty of Medicine, Tel Aviv University, Tel Aviv, Israel; 2 Gertner Institute for Epidemiology and Health Policy Research, Sheba Medical Center, Ramat Gan, Israel; 3 Nursing Department, School of Health Sciences, Ashkelon Academic College, Ben Tzvi 12, 78211 Ashkelon, Israel

**Correction to: BMC Public Health 22, 1420 (2022)**

**https://doi.org/10.1186/s12889-022-13795-5**

The original publication of this article [1] contained a duplicated author: **Mor Saban**. The article has been updated to remove this duplication.
