# Supplementary material for: Teachers' characteristics predict students' guidance for healthy lifestyle: a cross-sectional study in Arab-speaking schools
Source: BMC Public Health. 2022 Jul 26;22:1420. doi: 10.1186/s12889-022-13795-5 (PMC9321300; doi:10.1186/s12889-022-13795-5)
Supplement: Supplementary file 1 — Additional file 1. [file 12889_2022_13795_MOESM1_ESM.docx]

**Supplementary file: English translation of the health behavior section of the teachers’ questionnaire**

**Do teachers lead a healthy lifestyle?**

Dear teacher

We would like to evaluate to which degree do teachers lead a healthy lifestyle. I kindly ask to dedicate time for completing a short questionnaire. Your privacy and confidentiality are guaranteed.

Sincerely yours

Roaa Kittany, Masters of Public Health student

1. **In a typical week, how many times do you perform moderate-intensity physical activity** such as brisk walking, body-building exercises, ball games, dance or yoga?

- Not at all
- 1
- 2
- 3
- 4
- 5
- 6
- 7

1. **On average, how long does each episode of activity last?** ______ minutes
2. **In a typical week, how many times do you perform vigorous-intensity physical activity** that causes considerable increase in respiratory and heart rates as well as profound sweating, such as running, swimming or rapid cycling?

- Not at all
- 1
- 2
- 3
- 4
- 5
- 6
- 7

1. **On average, how long does each episode of activity last?** ______ minutes

**Which of the following questions (numbers 5-10) best describes your nutrition and eating habits?**

1. **Eating breakfast**

- Daily or almost daily
- 3-4 times a week
- 1-2 times a week
- Less than once a week
- Not at all

1. **Adherence to the principles of a Mediterranean diet**: intake of vegetables, fruit, legumes, olive oil, nuts, unrefined wheat, fish, poultry and low-fat dairy products.

- Daily or almost daily
- 3-4 times a week
- 1-2 times a week
- Less than once a week
- Not at all

1. **Drinking 8 cups of water per day**

- Daily or almost daily
- 3-4 times a week
- 1-2 times a week
- Less than once a week
- Not at all

1. **Eating processed food products**

- Daily or almost daily
- 3-4 times a week
- 1-2 times a week
- Less than once a week
- Not at all

1. **Drinking sugar-sweetened beverages, including fruit juices**

- Daily or almost daily
- 3-4 times a week
- 1-2 times a week
- Less than once a week
- Not at all

1. **Eating 5 units of fruits and/or vegetables per day**

- Daily or almost daily
- 3-4 times a week
- 1-2 times a week
- Less than once a week
- Not at all

1. **In the last month, how many hours, on average, did you sleep at night?**

- 5 or less
- 6
- 7
- 8 or more

1. **In general, how would you define your health status?**

- Excellent
- Very good
- Good
- Fair
- Poor

1. **To what extent do you experience emotional stress?**

- Very low
- Low
- Moderate
- High
- Very high

1. **Do you undergo the periodic age-recommended screening tests?**

- not at all
- partially
- fully as recommended

1. **For the calculation of body mass index (BMI)**

- Your height in centimeters is_____ (according to the last measurement that you can recall)
- Your weight in kilograms is______ (according to the last time you stood on the scales)
